# Nonimmunologic urticaria due to prostaglandin E1

**DOI:** 10.1016/j.jdcr.2026.07.040

**Published:** 2026-07-30

**Authors:** Daphne Pate, Akshitha Thatiparthi, Kiran Motaparthi, Pooja Gurnani

**Affiliations:** a Wayne State University School of Medicine, Detroit, Michigan; b University of Florida Department of Dermatology, Gainesville, Florida

**Keywords:** alprostadil, neonatal rash, nonimmunologic urticaria, prostaglandin E1

## Introduction

Prostaglandin E1 (PGE_1_) analogs are routinely used for maintaining ductal patency in neonates with ductal-dependent congenital heart disease (CHD), until definitive surgical treatment. While systemic adverse effects (AEs) are well reported, only a few reports have described cutaneous eruptions associated with PGE_1_ administration.^1^^,^^2^ Often mistaken for hypersensitivity reactions, these eruptions instead represent nonimmunologic, dose-dependent urticarial responses. We present a case of a transient, urticarial eruption temporally associated with PGE_1_ infusion. Recognition of PGE_1_-associated cutaneous eruptions is important in clinical practice to prevent unnecessary interruption of life-sustaining therapy in neonates with ductal-dependent CHD.

## Case report

**Fig 1:**
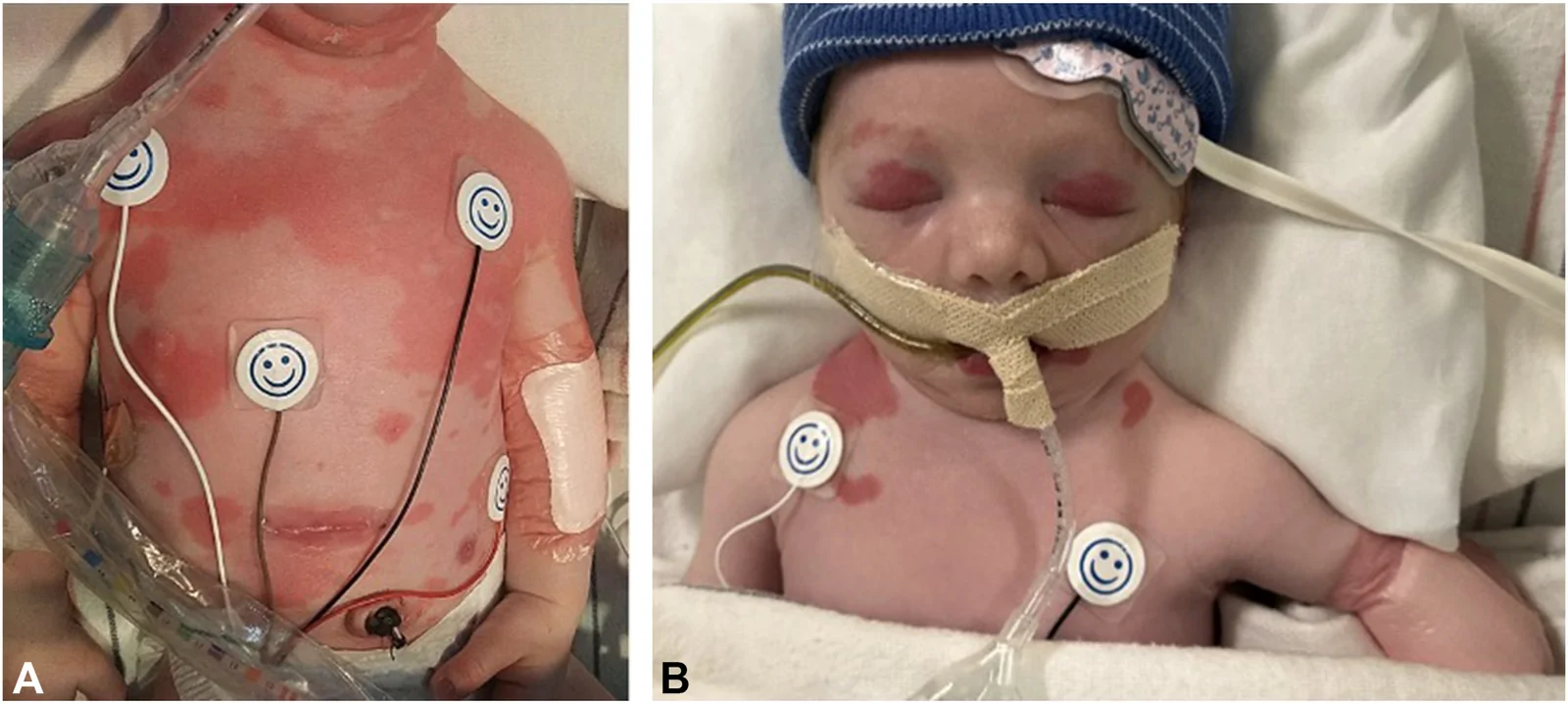
Nonimmunologic urticaria due to prostaglandin E1. Migratory erythematous patches on the trunk **(A)**, proximal extremities, and face **(B)** of a neonate receiving alprostadil infusion.

A 9-day old male, born at 37 weeks and 6 days to a healthy 30-year-old G2P2002 mother after an uncomplicated pregnancy and spontaneous vaginal delivery, was admitted to the neonatal intensive care unit due to double outlet right ventricle and Tetralogy of Fallot with large ventral septal defect, right ventricular hypertrophy, pulmonic valve stenosis, and overriding aorta. A continuous alprostadil infusion was initiated on day 2 of life at 0.3 mcg/kg/min × 2.9 kg (2.61 mL/h) to maintain ductal patency. Dermatology was consulted on day 9 of life for a generalized erythematous eruption. On physical exam, migrating, annular and polycyclic erythematous patches were noted on the face, trunk and extremities (Fig 1, *A* and *B*), with individual lesions resolving over several hours. No extracutaneous manifestations were present, and no signs or symptoms consistent with anaphylaxis were observed. The differential diagnosis included erythema toxicum neonatorum and viral exanthem, neither of which carries a temporal relationship to drug administration. Given the timing and medication history, age of the neonate, and morphology, the eruption was consistent with a PGE_1_-induced, nonimmunologic urticarial eruption. Diphenhydramine provided mild improvement but not resolution. Topical therapies were not administered. Alprostadil was continued at 0.32 mcg/kg/min × 2.74 kg (2.63 mL/h) to maintain ductal patency, with the rash persisting throughout the 11-day treatment course. Serum tryptase level was found to be 2.3 ug/L (normal). The patient’s TOF repair with PFO creation and VSD closure with transannular patch and infundibular resection was performed successfully, with resolution of the rash within 1 hour after discontinuation of PGE_1_. No recurrence was noted on follow-up at 20 days after discontinuing alprostadil.

## Discussion

Alprostadil, a synthetic PGE_1_ analog (PGE_1_ a-cyclodextrin clathrate) and potent vasodilator, is routinely used in the treatment of ductal-dependent CHD to maintain patency of the ductus arteriosus prior to surgical repair.^2^ Reported AEs of PGE_1_ include hypokalemia, hypotension, apnea, bradycardia, heart failure, hyperthermia, Pseudo-Barett syndrome, and gastric outlet obstruction.^2^ However, cutaneous reactions are rarely reported, with only 11 total reports in the literature (Table I).

**Table I:** Cutaneous manifestations due to prostaglandin E1 administration

Author/year	Sex/age	Indication for PGE_1_	Cutaneous presentation	Biopsy/treatments	Course/outcome
Urticaria					
Carter & Garzon (2000)^1^	Two-day old male.	TGA with small PFO.	Eruption of polycyclic and iridial patches of blanching erythema on the scalp, neck, chest, and left ankle.	Biopsy showed sparse mononuclear infiltrate.	Dose dependent erythema and surface area. Full resolution intra-operatively after PGE_1_ stopped with no recurrence.
Young et al (2016)^3^	Two-day old male.	Dilated right-sided cardiac structures and a large PDA with right-to-left shunt.	Brightly erythematous annular and polycyclic patches on the face, trunk, and extremities, which migrated throughout the day, sometimes within minutes.	No biopsy. Not responsive to diphenhydramine.	Complete resolution of the rash within minutes of discontinuation, then immediate recurrence of the rash when restarted. No recurrence after day 8 of life.
Wheless et al (2017)^4^	Two-month-old male.	Complex CHD awaiting a heart transplant on venoarterial ECMO.	Generalized rapidly migratory erythematous patches with sharp demarcation of erythema at the distal tips of the fingers and toes.	No biopsy. Temporal association with PGE_1_.	Patient had been on PGE_1_ for almost 2 months. Rash did not appear until the dose was increased after hypoxia and placed on venoarterial ECMO. Resolved with decannulation.
Ruknoo et al (2020)	Two- day old male.	Taussig-Bing anomaly, d-TGA, large subpulmonic VSD, large PDA, severe coarctation and interrupted aortic arch.	Migratory polycyclic erythema of urticaria of the face, scalp, neck, and head.	No biopsy. Dose dependent relationship noted.	Resolved with decreasing dose of PGE_1_ to 0.01 mcg/kg/min.
Daugherty et al (2021)	One-day old female.	TGA.	Migratory, annular, blanching, erythematous patches on her face, scalp, neck, and trunk.	Marked vascular ectasia, mild superficial dermal edema, minimal dermal inflammation, and sparse intravascular neutrophils. Unresponsive to diphenhydramine.	Resolved rapidly upon discontinuation of PGE_1_.
Raman et al (2022)^5^	Ten-day old male.	Hypoplastic left heart syndrome on ECMO.	Transient, migratory, ill-defined, bright pink, blanchable patches on the left forehead and periocular skin and polycyclic, bright red, edematous plaques without overlying scale on the left chest and upper arm.	No biopsy. Described classic features of sparse mononuclear infiltrate (Carter, 2000).	Resolved within days after discontinuation of PGE_1_.
Shah et al (2024)	Two-day old female.	DORV with critical pulmonary valve stenosis, threatened discontinuity of the left pulmonary artery, and tortuous PDA.	Migratory bright erythematous annular and polycyclic patches with angulated and well-defined circular and geographic borders were noted on the face, trunk, and extremities.	No biopsy.	Complete resolution 5 minutes after PGE_1_ was decreased to 0.03 mcg/kg/min.
Önen Ocak et al (2025)	Three-day old male.	Severe right ventricular hypoplasia, a hypoplastic right AV valve, d-TGA, hypoplasia of the ascending aorta and aortic arch, a secundum ASD, a subpulmonic VSD, and a large PDA.	Multiple erythematous eruptions starting as small, raised pink-to-red lesions on the trunk, face, and scalp then enlarging to form a ring shape with a central clearing.	No biopsy. Dose dependent relationship observed.	Resolved intraoperatively with PGE_1_ discontinuation.
Rao et al (2004)^6^	Case I: Ten-day old female. Case II: 8-day old female.	Case I: TGA. Case II: Pulmonary atresia.	Case I and II: Blanchable migratory, macular erythema on head, neck, and trunk with midline demarcation.	Case I and II: No biopsy. Unresponsive to diphenhydramine and topical hydrocortisone.	Resolved with discontinuation of PGE_1_.
Tsuboi et al (2021)	Eleven-day old male.	Persistent pulmonary hypertension of the neonate on ECMO.	Macular, blanchable erythema developed on the head, neck and half of the trunk with sharp demarcation along the midline.	No biopsy. Negative bacterial workup.	Recurrent episodes persisted throughout his course until death at day 30.
Panigrahi et al (2024)^7^	Three-day old male.	TGA with shunts, ASD, and PDA.	Erythematous blanchable rash involving the scalp, face, and upper trunk with sharp demarcation between the upper and lower abdomen.	No biopsy.	Resolution with decreasing dose to 20 ng/kg/min. No further recurrence.

*ASD*, Atrial septal defect; *AV valve*, atrioventricular valve; *CHD,* congenital heart disease; *d-TGA,* dextro-transposition of the great arteries; *DORV,* double outlet right ventricle; *ECMO*, extracorporeal membrane oxygenation; *PDA*, patent ductus arteriosus; *PFO*, patent foramen ovale; *PGE*_*1*_*,* prostaglandin E1; *TGA*, transposition of the great arteries; *VSD*, ventricular septal defect.

While conventional urticaria shares a similar morphology of transient irregular wheals, this case differs in that the eruption was annular/polycyclic and migratory. The eruptions also had a close temporal relationship with alprostadil administration and normal serum tryptase level. Across reported cases of PGE_1_ induced cutaneous urticarial eruptions, the timing, morphology, and pattern of resolution were consistent. The rash begins within hours to days of starting PGE_1_ infusion as a migratory, blanchable, asymptomatic eruption. Most had an annular or polycyclic morphology, while others were geometric. All cutaneous eruptions demonstrated dose-dependent changes with PGE_1_ administration and resolved completely with discontinuation.

The dose-dependent nature of the urticarial eruption further supports a nonimmunologic mechanism, as IgE-mediated hypersensitivity reactions do not typically demonstrate predictable changes in severity with increasing drug exposure.^3^^,^^6^ The typical lack of response to antihistamines further indicates an immunoglobulin E–independent pathway.^3^^,^^6^ Prostaglandin E_1_ induces this urticarial eruption through direct activation of mast cells and basophils, stimulating cyclic adenosine monophosphate (cAMP) production and potassium-channel activation, resulting in the degranulation of histamine.^7^^,^^8^ The resulting vasodilation and increased capillary permeability clinically manifest as transient, blanchable, polycyclic erythema.^1^^,^^3^ Intradermal PGE_1_ produces a dose-dependent wheal-and-flare reaction where antihistamines suppress the wheal but not the flare, suggesting that histamine is not the sole mediator and that components of the cutaneous response are independent of histamine signaling.^8^ Other pathways suggested include bradykinin-induced angioedema, activation of the coagulation cascade, and increased levels of neuropeptide substance P.^1^^,^^3^^,^^5^ Additionally, serum tryptase level was measured in this patient and found to be normal. This finding argues against severe systemic mast cell activation, red man syndrome associated with vancomycin, or anaphylaxis, but does not exclude PGE1-induced nonimmunologic urticaria. Therefore, tryptase levels may provide supportive diagnostic evidence in distinguishing PGE1-induced urticaria from other differential diagnoses.^9^

Some patients described in the literature were on venoarterial extracorporeal membrane oxygenation (ECMO). In a patient on venoarterial ECMO, the metabolism of PGE_1_ may be altered from decreased pulmonary blood flow. Up to 80% of circulating PGE_1_ is metabolized by the lungs and is inactivated during first-pass pulmonary circulation.^4^^,^^10^ In the context of reduced pulmonary blood flow, even stable infusion rates can yield excessive vasodilatory activity. Hypoxia may enhance endothelial responsiveness to prostaglandins, amplifying vasodilation and vascular leakage.^1^^,^^3^ Wheless et al, reported a patient on a PGE_1_ infusion for 2 months, but only developed a cutaneous reaction once the patient experienced hypoxia and was placed on ECMO.^4^ Both impaired pulmonary metabolism and tissue hypoxia potentiate the same nonimmunologic pathway, explaining why eruptions can arise or worsen in hypoxic infants despite stable infusion rates.

Recognition of this urticarial eruption due to PGE_1_ administration has important clinical implications for neonatal and dermatologic management. Misinterpretation may lead to unnecessary interventions, including systemic steroid exposure, antibiotic use, or premature discontinuation of therapy, potentially jeopardizing hemodynamic stability. Prostaglandin E_1_ is lifesaving for infants with cyanotic CHD, and therefore, should not be discontinued unless necessary. Given that histopathology demonstrates nonspecific edema and sparse to absent inflammation, skin biopsy is not necessary for diagnosis and should be avoided.^1^ Management relies on supportive care and titration to the lowest effective dose and infusion rate to maintain ductal patency. Corrective surgery for the patient’s CHD should not be delayed due to this urticarial eruption. The eruption is self-limited and resolves rapidly after dose adjustment in all cases, highlighting that further intervention is unnecessary.

## Conflicts of interest

None disclosed.
